# CabHLH18-like Gene Promotes Leaf Yellowing and Directly Activates *CaCLH1* in Pepper

**DOI:** 10.3390/plants15172627

**Published:** 2026-08-28

**Authors:** Zhanghong Yu, Zhe Zhang, Luokun Rong, Linbin Yan, Yaning Meng

**Affiliations:** 1 Institute of Cash Crops, Hebei Academy of Agriculture and Forestry Sciences, Shijiazhuang 050051, China; yzhong2021@163.com (Z.Y.); luanzhi914@163.com (Z.Z.); rongluokun@163.com (L.R.); tianjiaoshi@126.com (L.Y.); 2 Hebei Province Engineering Research Center for Vegetables, Shijiazhuang 050051, China

**Keywords:** yellow leaf, *CabHLH18-like* gene, *CaCLH1*, chlorophyll accumulation, pepper

## Abstract

Yellow leaf phenotypes represent an important trait in pepper and enrich genetic germplasm resources. Previous studies have primarily examined leaf yellowing caused by defects in chlorophyll biosynthesis, whereas the functions of chlorophyll degradation-related genes remain less well characterized. In this study, a yellow leaf phenotype (NY) was identified. Transmission electron microscopy and RNA-seq showed that differentially expressed genes were concentrated in photosynthesis- and chlorophyll-related pathways. *CaCLH1* was then demonstrated to promote chlorophyll degradation. Yeast one-hybrid analysis identified the CabHLH18-like gene as a direct interactor with the *CaCLH1* promoter and as a positive regulator of *CaCLH1* transcription. The *CabHLH18-like* gene was highly expressed in yellow leaf pepper. The *CabHLH18-like* gene was subsequently isolated and characterized, revealing nuclear localization and transcription factor activity. Functional analysis showed that silencing the *CabHLH18-like* gene increased chlorophyll content, whereas its overexpression reduced chlorophyll content. These findings indicate that *CaCLH1* is a direct downstream target of the *CabHLH18-like* gene and contributes to *CabHLH18-like*-gene-mediated regulation of leaf yellowing in pepper. This study refines mechanistic understanding of leaf yellowing and provides a foundation for future research and breeding applications in pepper.

## 1. Introduction

Sweet pepper (*Capsicum annuum* L.) is cultivated worldwide for its diverse morphology, vibrant coloration, high economic value, and broad application potential [1,2]. Leaves, which respond rapidly to biotic and abiotic stresses, serve as key indicators in pepper breeding and cultivation. As a high-value vegetable crop, sweet pepper depends strongly on photosynthetic efficiency for yield and fruit quality, so leaf functional status is a critical determinant of agricultural productivity. Leaf color is a visually accessible and physiologically informative trait that reflects chlorophyll metabolism and photosynthetic capacity, and it is widely used to assess plant development, stress responses, and genetic variation. Accordingly, elucidating the genetic and molecular basis of leaf color variation in pepper provides fundamental insight into chloroplast biology and offers practical tools for early-stage selection of vigorous genotypes and stress-resilient cultivars, thereby directly supporting breeding programs aimed at improving crop performance.

Color variation in leaves is a widespread and visually striking trait in plants. Leaf color mutants commonly display phenotypes such as albinism, light green, dark green, or variegation, which are readily recognizable. These phenotypic changes arise mainly from impaired chlorophyll biosynthesis or accelerated chlorophyll degradation. Leaf color is largely governed by chlorophyll and carotenoids, with chlorophyll predominating and imparting the characteristic green hue through selective light reflection. Chlorophyll is a magnesium (Mg)-porphyrin compound, and its biosynthesis is a hallmark of photomorphogenesis that signals the onset of autotrophic growth. It is therefore central to both leaf coloration and overall plant development [3]. Chlorophyll a is synthesized from glutamate through a defined series of intermediates including δ-aminolevulinic acid (ALA), porphyrin III, Mg-protoporphyrin IX, and chlorophyllide a. This biosynthetic pathway is catalyzed by key enzymes such as ALA dehydratase, protoporphyrinogen oxidase, Mg-chelatase (MgCh), and chlorophyll synthase (*HEMA*, *PPO*, *CHLI*, *CHLG*) [4,5,6], and it ultimately establishes green leaf pigmentation [7]. In *Arabidopsis thaliana*, silencing glutamyl-tRNA reductase 1 (*HEMA1*) reduces 5-aminolevulinic acid (ALA) content and chlorophyll levels [8,9]. Mg-chelatase (MgCh) is a heterotrimeric complex of CHLI, CHLD, and CHLH subunits that catalyzes magnesium insertion into protoporphyrin IX and thereby executes as a pivotal commitment step in chlorophyll biogenesis. In the rice *chlorina-1* mutant, a missense mutation of *CHLD* lowers Mg-protoporphyrin IX content and suppresses chlorophyll accumulation [10]. Similarly, silencing *CHLI* in *Arabidopsis thaliana* compromises MgCh complex abundance and activity and leads to leaf yellowing [11], whereas mutation of *BrCHLD* produces comparable phenotypes in Chinese cabbage [12].

Chlorophyll homeostasis is modulated by both environmental and endogenous signals, including light, circadian rhythms, and abiotic stresses. Golden2-like (GLK) transcription factors preserve leaf greenness by promoting chloroplast development and plastid proliferation [13,14]. Phytochrome-interacting factors (PIFs) regulate chloroplast photomorphogenesis and thus affect leaf coloration. In *A. thaliana*, analysis of the *pif3* mutant has demonstrated that *AtPIF3* represses chlorophyll biosynthesis genes, including *HEMA1*, *GUN5*, *LHCA1*, and *PsaE1*, which in turn influences leaf color [15]. HY5, a central regulator of light signaling, regulates chloroplast development and chlorophyll biosynthesis by binding the promoter of *GLK2* in *Arabidopsis*, thereby sustaining a stay-green phenotype under light conditions [16]. WRKY transcription factors function in both chlorophyll biosynthesis and degradation. In Chinese cabbage, *WRKY22* and *WRKY53* activate *CHLP* and *CHLH* expression to maintain chlorophyll accumulation under cold stress [17,18], whereas in citrus, *WRKY42* induces the expression of *NYC* and *SGR*, accelerating degreening and fruit yellowing [19].

Leaf coloration is also affected by chlorophyll degradation. Chlorophyllase (CLH) catalyzes the hydrolysis of chlorophyll by removing the phytol side chain, thereby generating chlorophyllide and phytol. It has long been considered the key initiating enzyme in the chlorophyll catabolic pathway. The *CLH* gene was first cloned from *Chenopodium album* and *Arabidopsis thaliana* in 1999, which revealed its lipase motif and it expression characteristics induced by methyl jasmonate (MeJA) [20]. Transient overexpression of *MdCLH1* in apple leaves significantly reduced chlorophyll a and total chlorophyll contents, while it increased chlorophyll b, indicating that *MdCLH1* regulates chlorophyll degradation and alters photosynthetic parameters [21]. VIGS-mediated silencing of *PsCLH1* in tree peony petals significantly increased chlorophyll content and enhanced green coloration, indicating that *PsCLH1* promotes chlorophyll degradation to control petal degreening [22].

Leaf color variation in pepper has largely been attributed to defects in chlorophyll biosynthesis, whereas the role of chlorophyll degradation has remained less clearly defined. In particular, the genetic basis of degradation-mediated leaf yellowing has received relatively limited attention. Here, we investigated a yellow leaf variant (NAIYOUJIAO, NY) and a normal green line (ZIXINGJIAO, ZX), focusing on pathways related to photosynthesis and chlorophyll metabolism. Given the central role of chlorophyll turnover in determining leaf pigmentation, we further examined candidate genes associated with chlorophyll degradation. We identified that *CaCLH1* positively regulates chlorophyll degradation in pepper. In addition, we identified the CabHLH18-like gene as an upstream regulatory gene of *CaCLH1*. Functional characterization of the *CabHLH18-like* gene demonstrated its critical role in leaf color determination. This work clarifies the contribution of chlorophyll degradation in shaping leaf color phenotypes in pepper and provides a framework for exploiting these traits in germplasm improvement and selection.

## 2. Results

### 2.1. Morphological Phenotype in Two Pepper Cultivars

**Figure 1:**
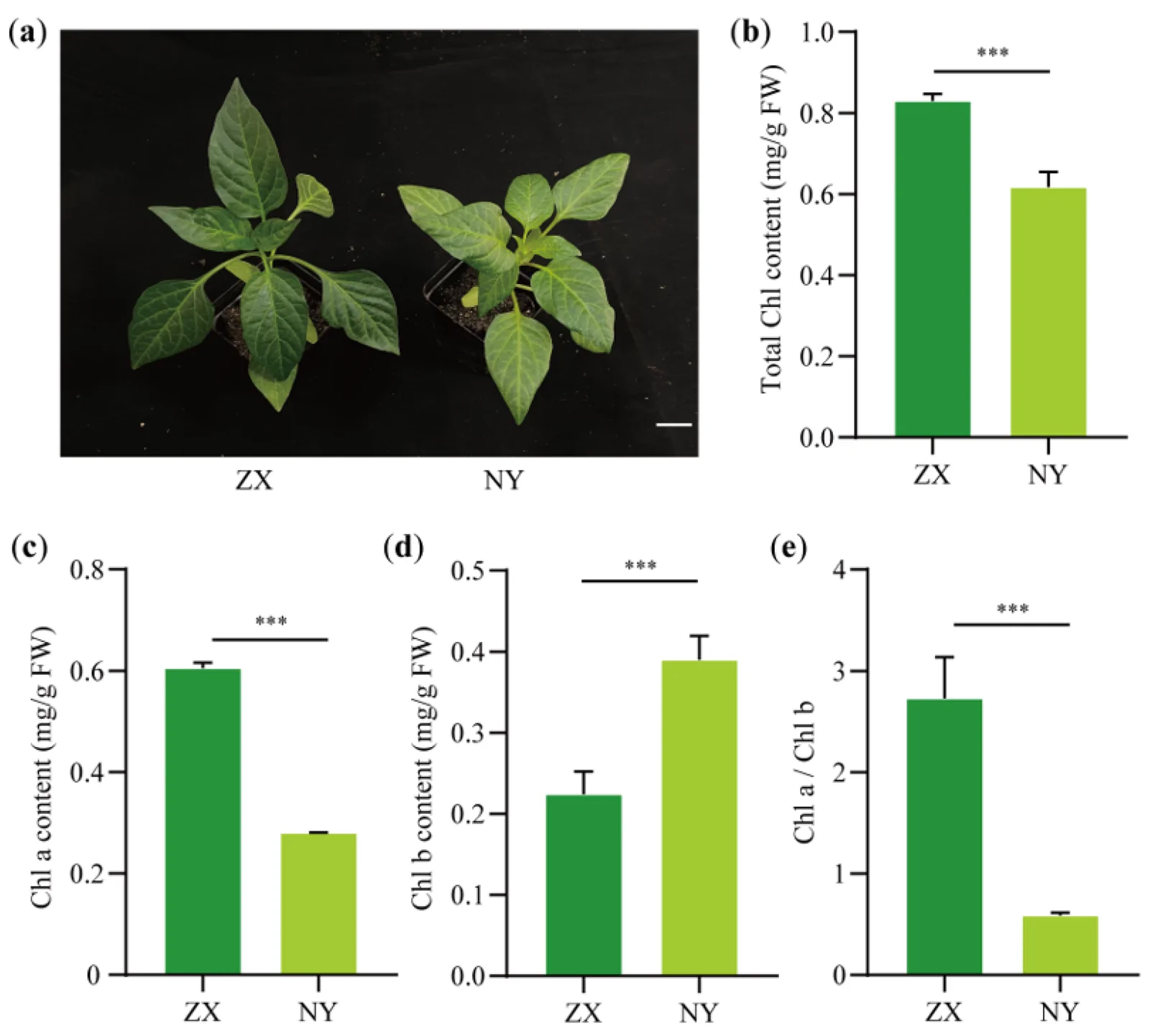
Phenotypic and chlorophyll differences between ZX and NY. (**a**) Leaf phenotype of ZX and NY. Scale bar = 2 cm. (**b**–**e**) Total chlorophyll content (Chl), Chl a content, Chl *b* content, and the Chl a/Chl b ratio. Statistical significance was established via Student’s *t*-test (*** *p* < 0.001).

Two 8-week-old sweet pepper cultivars with contrasting leaf phenotypes were identified: ZX (green leaves) and NY (yellow leaves) (Figure 1a). Little differences in chloroplast ultrastructure were detected between the two cultivars (Figure S1). To assess their pigment composition, chlorophyll (Chl) content was quantified. ZX accumulated more total chlorophyll than NY (Figure 1b). Chl a content was lower in NY leaves than in ZX (Figure 1c), whereas Chl b content was higher in NY (Figure 1d). As a result, the Chl a/Chl b ratio was higher in ZX than in NY (Figure 1e). The reduced chlorophyll a/b ratio in NY indicated a marked increase in chlorophyll b content, which may reflect either compensatory enlargement of the light-harvesting antenna complex (LHC) or enhanced chlorophyll catabolism in NY. These differences in chlorophyll composition are likely linked to the contrasting variation in leaf coloration between the two cultivars.

### 2.2. Transcriptomic and Differential Gene Expression Analyses

**Figure 2:**
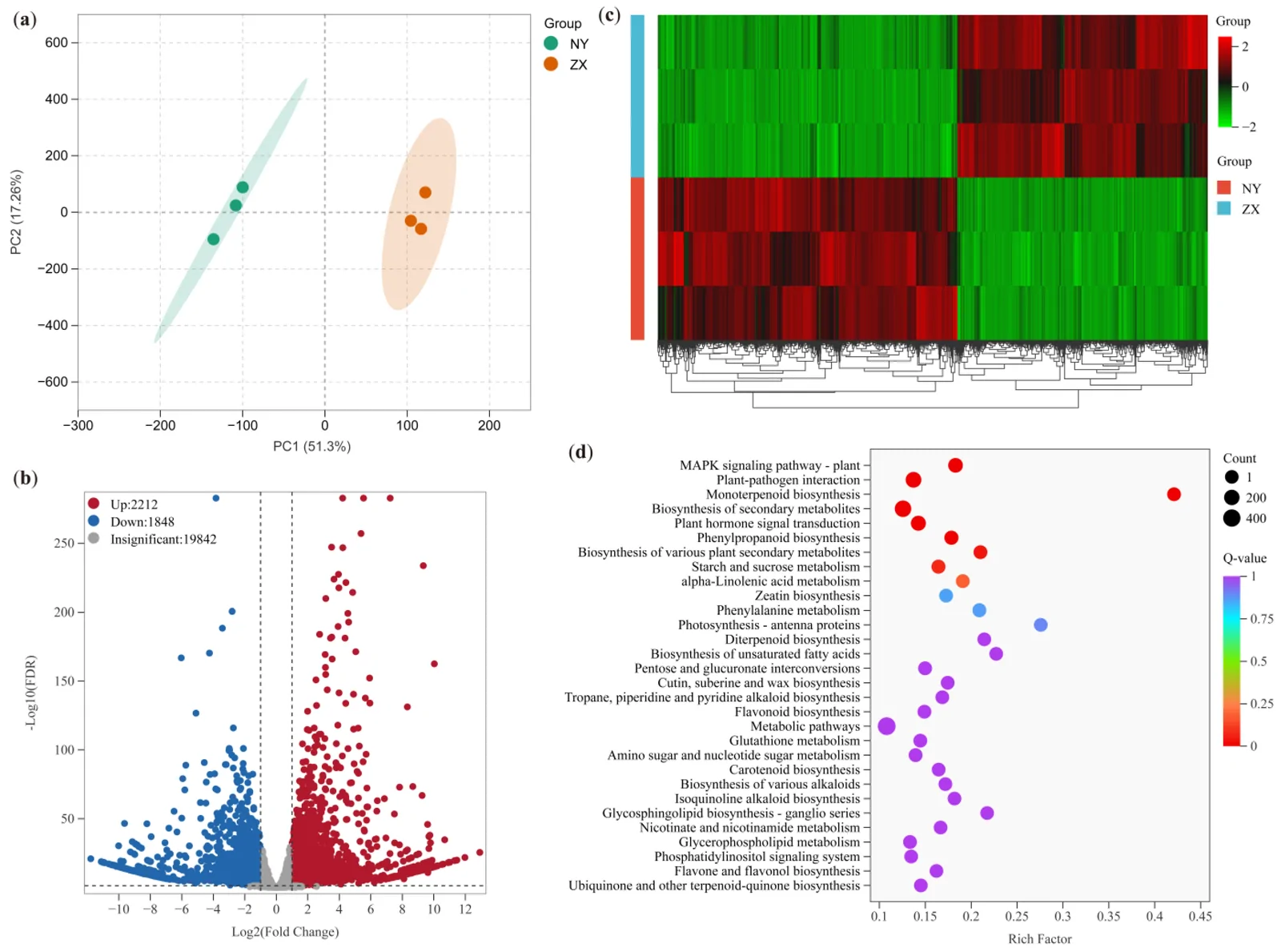
Transcriptome analysis in pepper. (**a**) PCA visualization for the full samples along PC1 (51.3% variance) and PC2 (17.26% variance). Green circles, yellow leaf replicates; red circles, green leaf replicates. (**b**) A volcano plot was constructed to visualize the DEGs between NY and ZX. (**c**) Heatmap of the DEGs. Colors represent Z-score scaled expression intensities (red: upregulated; green: downregulated). Hierarchical clustering was performed using the Euclidean distance metric and the complete linkage method. (**d**) KEGG enrichment map of DEGs.

The two cultivars were subjected to transcriptomic sequencing. The result of principal component analysis (PCA) revealed clear separation between the green leaf and yellow leaf pepper samples along PC1 (51.3% variance) (Figure 2a). PC2 explained 17.26% of the variance and primarily reflected within-group variation. Notably, biological replicates within each genotype clustered tightly together, indicating high reproducibility and reliable detection of genotype-specific expression profiles. Of the 23,902 statistically tested genes, 4060 were deferentially expressed between NY and ZX, including 2212 upregulated and 1848 downregulated genes (Figure 2b). And then, the heatmap was identified and divided into two clusters: Cluster I contained genes upregulated in yellow-leaf samples, including regulatory transcription factors and chlorophyll degradation genes (*MYB*, *bHLH*, *CLH1*, *SGR*); Cluster II comprised genes downregulated in yellow leaves, predominantly chlorophyll biosynthesis genes and photosystem-associated genes (*CHLH*, *PORA*, *DVR*, *LHCI*) (Figure 2c).

These DEGs were classified into three major GO categories: biological process (BP), cellular component (CC), and molecular function (MF). As shown in Figure S2, 15 enriched terms were identified in BP, 2 in CC, and 15 in MF. KEGG pathway enrichment analysis was then conducted. The top 20 enriched pathways included the MAPK signaling pathway plant, monoterpenoid biosynthesis, plant hormone signal transduction, phenylpropanoid biosynthesis, and starch and sucrose metabolism. Pathways related to chlorophyll metabolism were significantly enriched, including photosynthesis (ko00195), carbon fixation in photosynthetic organisms (ko00710), and porphyrin metabolism (ko00860) (Figure 2d). These results indicate that variation in leaf color may be linked to changes in chlorophyll biosynthesis and metabolism.

### 2.3. qRT-PCR Validation of DEGs

**Figure 3:**
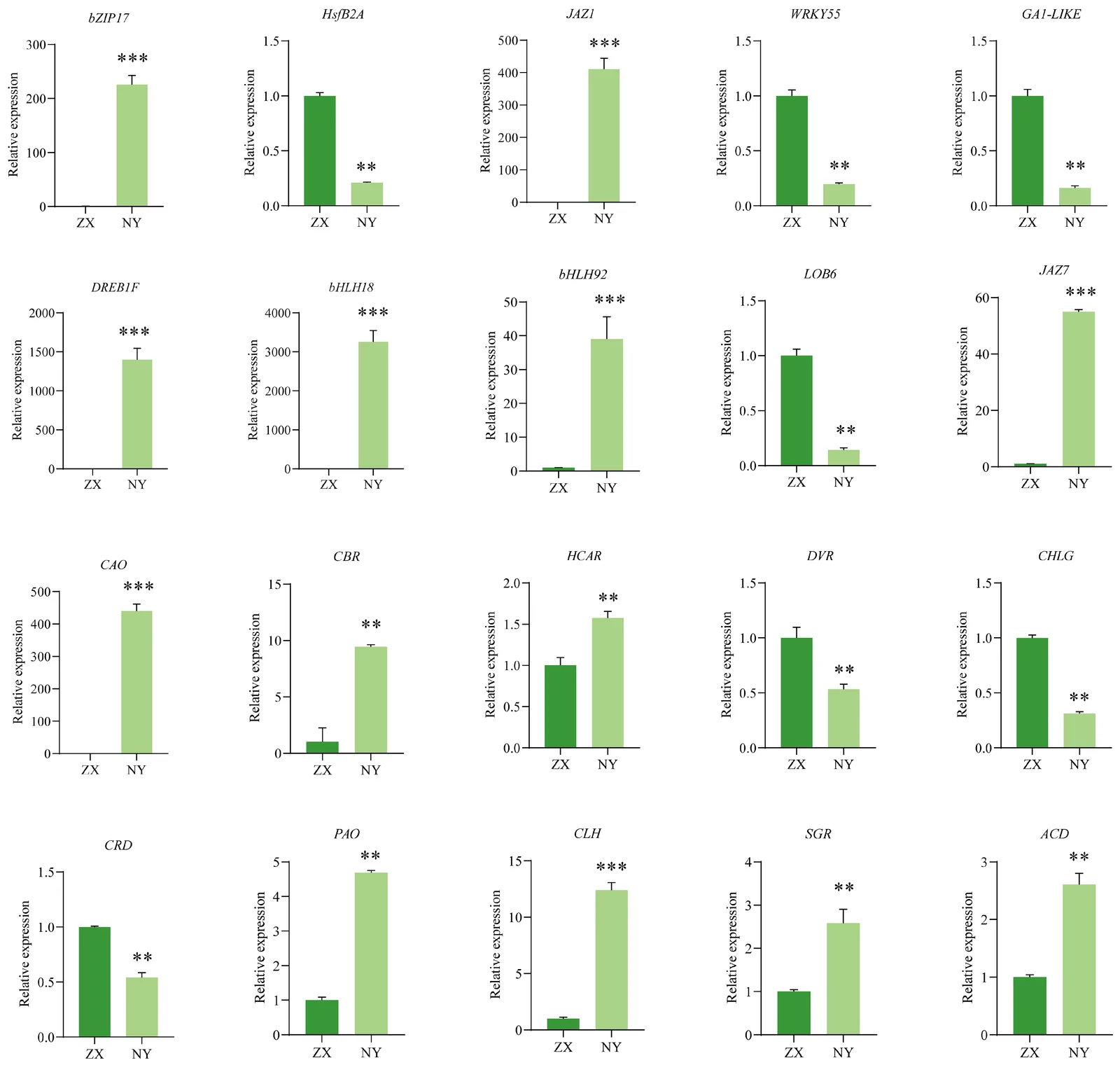
qRT-PCR validation of RNA-seq data. *CaActin* was used as the internal reference gene. Statistical significance was detected through Student’s *t*-test (** *p* < 0.01, *** *p* < 0.001).

Then, qRT-PCR was used to verify the reliability of the transcriptomic data. DEGs, including both upregulated and downregulated candidates, were randomly selected for validation. These genes were classified into four groups: chlorophyll-related transcription factors and genes involved in chlorophyll biosynthesis, chlorophyll cycling, and chlorophyll degradation. The expression patterns were consistent with the transcriptome data and thus confirmed the reliability of the sequencing results for subsequent analyses (Figure 3).

The expression patterns of the chlorophyll-related genes exhibited differed markedly between the two cultivars. Members of the *bHLH*, *bZIP*, and *JAZ* transcription factor families were significantly upregulated in the yellow pepper, whereas the *HsfB2A*, *WRKY55*, *GA1-like*, and *LOB6* genes showed higher expression in the green pepper. Among chlorophyll biosynthesis-related genes, *CRD*, *DVR*, and *CHLG* were significantly downregulated in NY compared to ZX, whereas *CAO*, *CBR*, and *HCAR* were upregulated (Figure 3). In addition, several chlorophyll metabolism-related genes, including *PAO*, *CLH*, *SGR*, and *ACD*, exhibited significantly higher expression in yellow leaves than in green leaves, with *CLH* showing approximately 6-fold higher expression. These results suggest that the leaf color differences between the two cultivars are closely associated with alterations in chlorophyll metabolism.

### 2.4. Overexpression of CaCLH1 Promotes Chlorophyll Degradation

**Figure 4:**
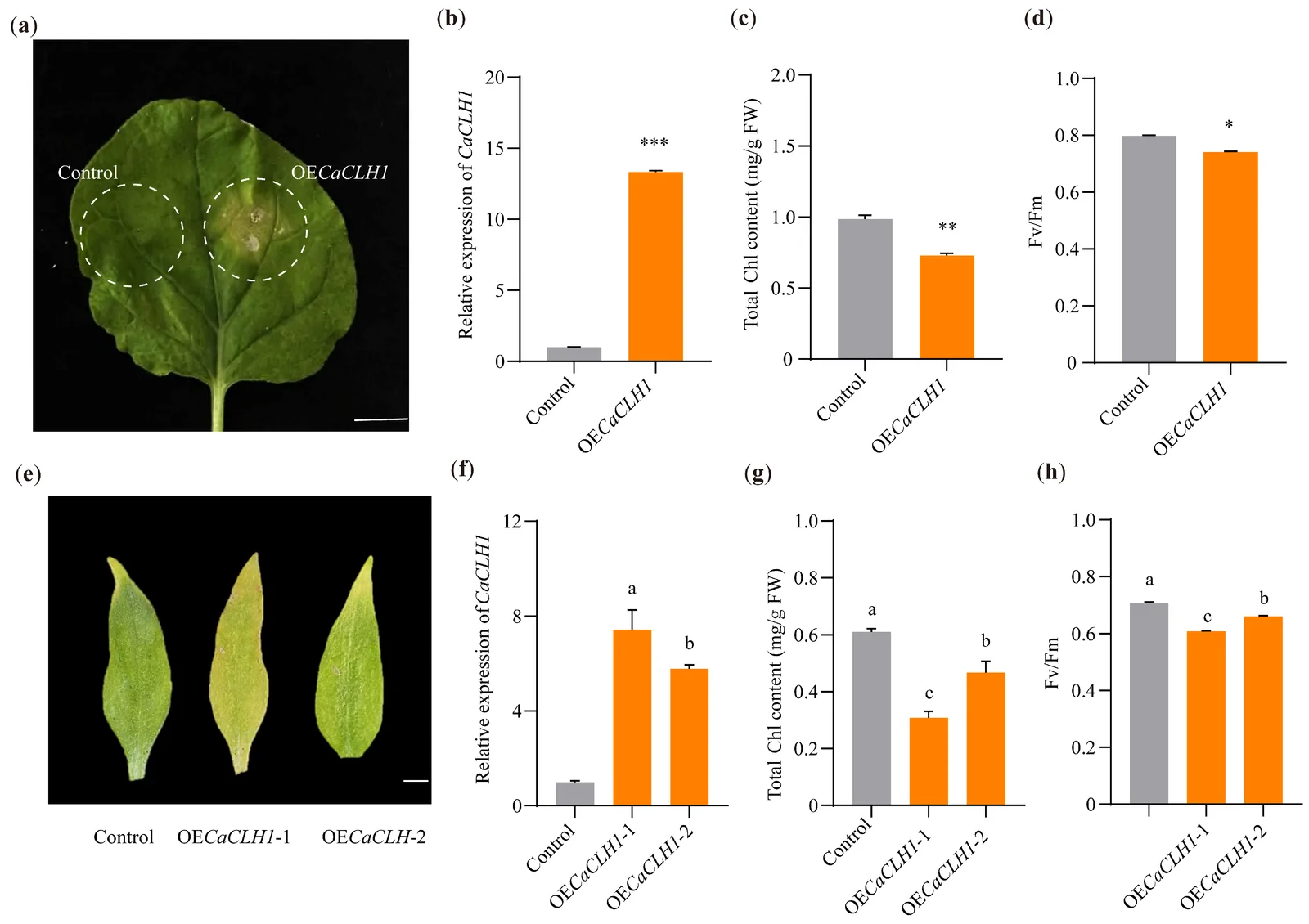
Transient overexpression of *CaCLH1* induced leaf yellowing in tobacco and pepper. (**a**,**e**) Phenotypic comparison of tobacco and pepper leaves infiltrated with *Agrobacterium tumefaciens* harboring pCambia1300-*CaCLH1*-GFP (OE*CaCLH1*) or empty vector pCambia1300-GFP (control). Bar = 1 cm. (**b**,**f**) Relative expression level of *CaCLH1* between control and *CaCLH1* transient expression leaves determined by qRT-PCR in tobacco and pepper. *CaActin* and *NbActin* served as the internal reference gene for pepper and tobacco, respectively. (**c**,**d**,**g**,**h**) Total chlorophyll content (**c**,**g**) and maximum quantum yield of photosystem II (*Fv*/*Fm*) (**d**,**h**) between control and *CaCLH1* transient expression leaves in tobacco and pepper. Mean ± SD, n = 3 biological replicates. Letters above the bars indicated statistically significant differences determined by one-way ANOVA (* *p* < 0.05, ** *p* < 0.01, *** *p* < 0.001).

In order to evaluate the function of *CaCLH1* in leaf coloration, *CaCLH1* was overexpressed in both pepper and tobacco. Transient overexpression assays showed that ectopic expression of *CaCLH1* in tobacco caused pronounced leaf yellowing and markedly reduced total chlorophyll content and maximum quantum yield of photosystem II (*Fv*/*Fm*) (Figure 4a–d). Meanwhile, *CaCLH1* was transiently overexpressed in pepper. Consistent with the results in tobacco, the transient-expression samples exhibited pronounced leaf yellowing (Figure 4e,f). The total chlorophyll and *Fv*/*Fm* were significantly decreased in *CaCLH1* transiently overexpressing leaves compared with the controls (Figure 4g,h). These results indicate that transient overexpression of *CaCLH1* promotes leaf yellowing and reduces chlorophyll content.

### 2.5. CabHLH18-like Binds to the Promoter of CaCLH1 to Activate Its Transcription

**Figure 5:**
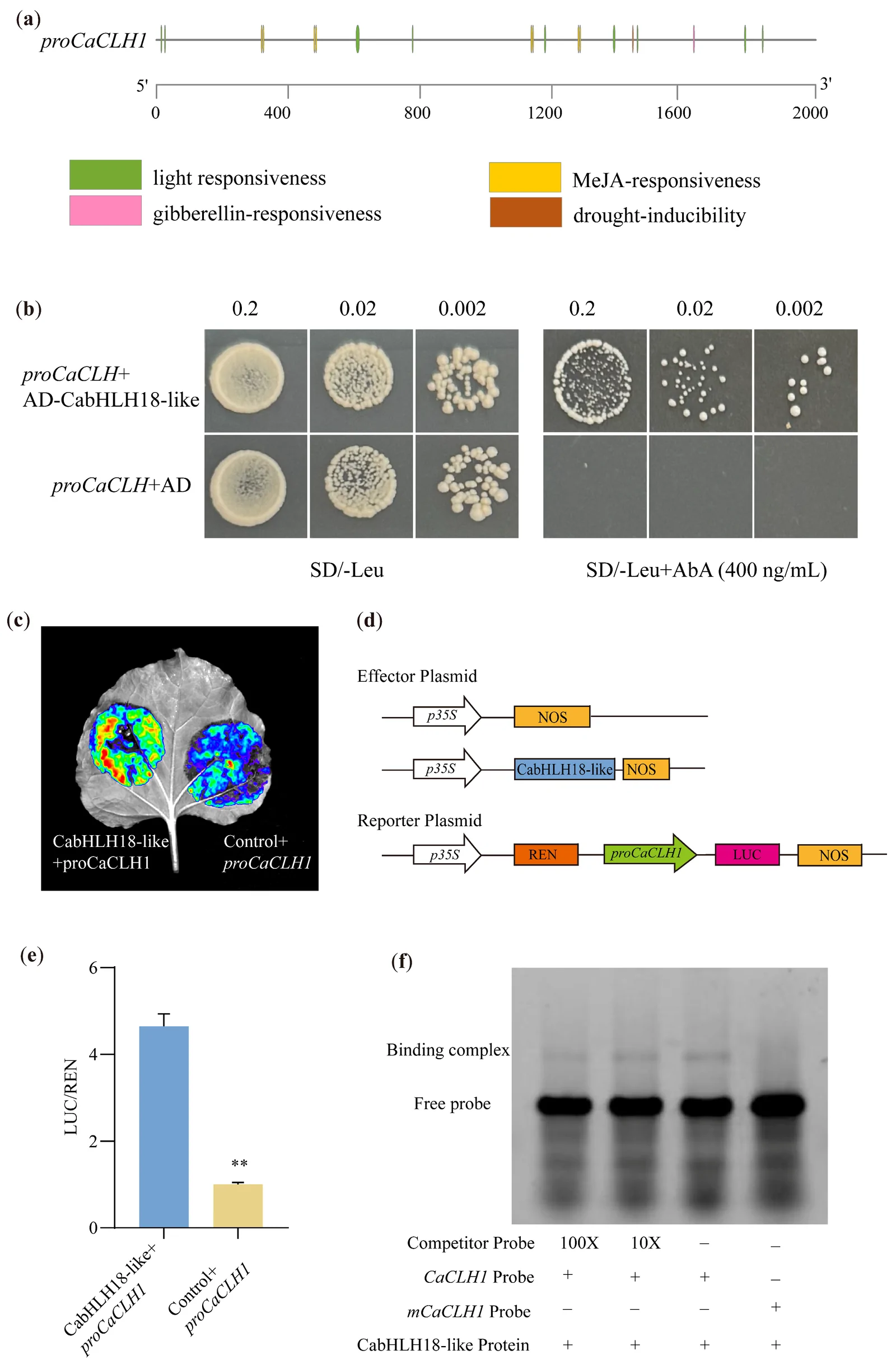
*CabHLH18-like* gene bound to the *CaCLH1* promoter to activate its transcription and promote chlorophyll degradation. (**a**) Cis-regulatory motif architecture of the promoter of *CaCLH1*. Putative binding motifs are indicated by colored boxes. (**b**) Yeast one-hybrid (Y1H) assay showing the interaction between the *CabHLH18-like* gene and the *CaCLH1* promoter. (**c**–**e**) LUC assays in Nicotiana benthamiana demonstrating that the *CabHLH18-like* gene activated the *CaCLH1* promoter transcription. Fluorescence images (**c**). Schematic of the effectors and reporter used (**d**). Relative LUC/REN ratios (**e**). (**f**) Electrophoretic mobility shift assay (EMSA) confirming the direct binding of recombinant *CabHLH18-like* protein to the (CANNTG) cis-element within the *CaCLH1* promoter. Biotin-labeled probes were incubated with purified his-*CabHLH18-like* protein; competition assays were performed with 10× and 100× unlabeled cold probes. For (**e**), data mined ± SD with three independent biological replicates. Student’s *t*-test: ** *p* < 0.01.

Based on RNA-seq data analysis and cis-element analysis of the *CaCLH1* promoter, we employed a yeast one-hybrid system to identify upstream regulatory genes of *CaCLH1* (Figure 5a). A bHLH family gene, the *CabHLH18-like* gene, was identified that could bind to the *CaCLH1* promoter (Figure 5b).

Dual-luciferase reporter assays (LUC) in tobacco leaves further confirmed that the CabHLH18-like gene strongly activates *CaCLH1* transcription, with a 2.8-fold increase in LUC/REN ratio compared with the empty effector control (Figure 5c–e). Electrophoretic mobility shift assays (EMSAs) demonstrated that specific binding of recombinant CabHLH18-like gene specifically binds to the E-box element within the *CaCLH1* promoter. The DNA–protein complex was effectively abolished by competition with unlabeled probes, whereas mutated probes failed to compete and thus confirmed the sequence-specific interaction (Figure 5f). Together, these results indicate that CabHLH18-like activates the transcription of *CaCLH1* by directly binding to its promoter.

### 2.6. Characterizations Pattern of the CabHLH18-like Gene

**Figure 6:**
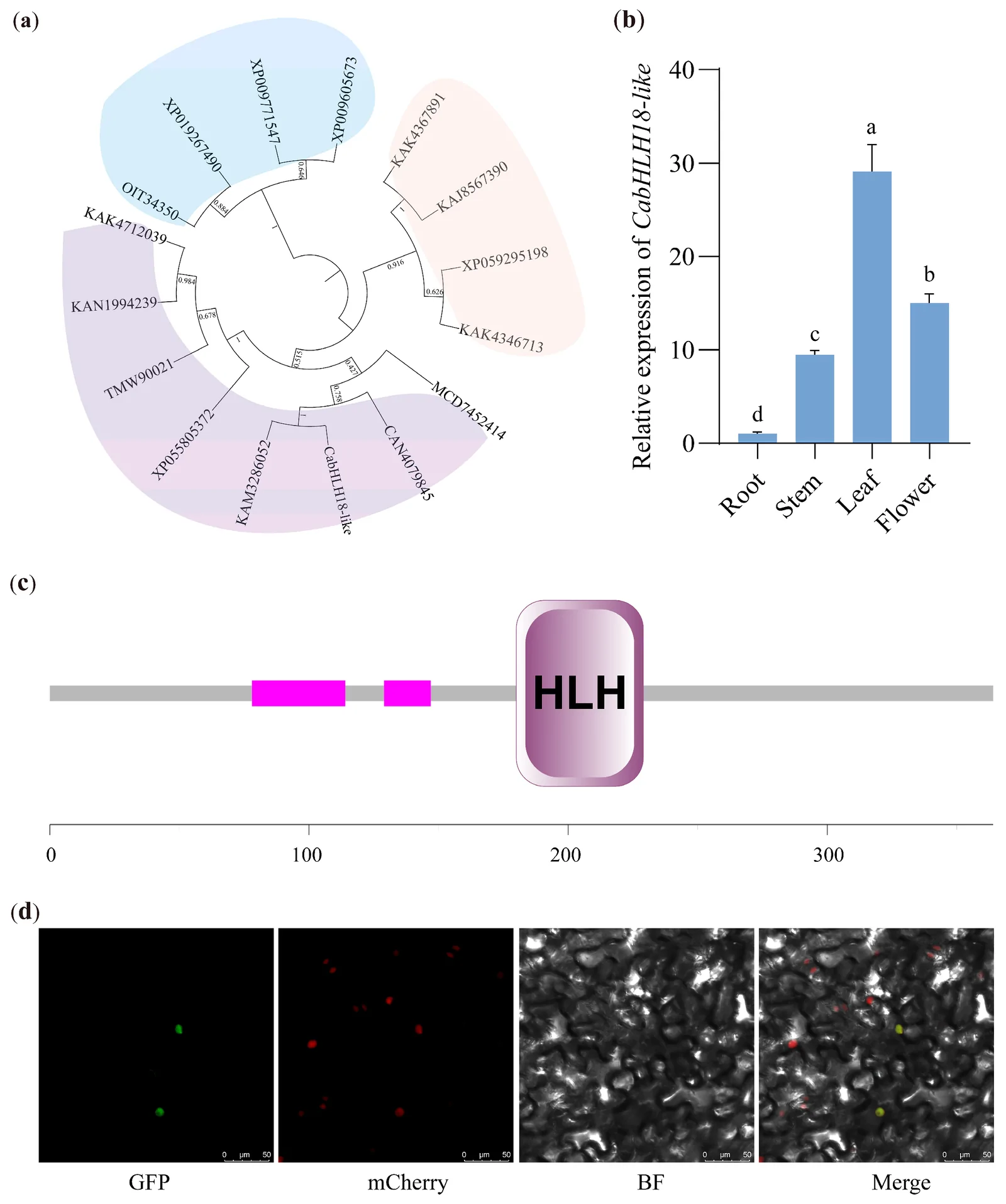
Expression pattern and subcellular localization of the *CabHLH18-like* gene. (**a**) Phylogenetic tree of the *CabHLH18-like* gene and its homologs from *Capsicum annuum*, *Capsicum chacoense*, *Solanum lycopersicum*, *Withania somnifera*, and other plant species. The neighbor-joining tree was constructed based on full-length amino acid sequences using MEGA X with the NJ model; bootstrap values from 1000 replicates were indicated at the nodes. The scale bar represented 0.5 substitutions per site. (**b**) Relative expression level of the *CabHLH18-like* gene in root, stem, leaf, and flower tissues of NY pepper. *CaActin* served as the internal reference gene. Mean ± SD from three independent biological replicates. Letters above the bars indicated statistically significant differences determined by one-way ANOVA followed by Tukey’s HSD test (*p* < 0.05). (**c**) Protein structure analysis of the CabHLH18-like gene. (**d**) Subcellular localization of the *CabHLH18-like* gene in *Nicotiana benthamiana*. GFP, green fluorescent protein signal; mCherry, nuclear marker signal; BF, bright field; Merge, overlay of GFP, mCherry, and BF channels. Scale bar = 50 μm.

Phylogenetic analysis showed that the *CabHLH18-like* gene and its homologs are grouped into three distinct clades (Figure 6a). The CabHLH18-like protein displayed the closest evolutionary relationship to its orthologs from *Withania somnifera* (CAN4079845) and *Capsicum chacoense* (KAM3286052), indicating strong evolutionary conservation of this gene across Solanaceae species.

Tissue-specific expression profiling showed that *CabHLH18-like* was highly expressed in leaves, with moderate expression levels detected in flowers and stems and minimal expression in roots (Figure 6b). This pattern suggests that the *CabHLH18-like* gene may primarily function in leaf development or physiology. Sequence analysis identified a conserved bHLH DNA-binding domain in the CabHLH18-like gene (Figure 6c). Subcellular localization analysis further demonstrated that the CabHLH18-like–GFP fusion protein was exclusively localized in the nucleus (Figure 6d), congruent with its inferred function as a DNA-binding regulator. Collectively, these results indicate that the *CabHLH18-like* gene is a nuclear-localized, evolutionarily conserved transcription factor with a potential tissue-specific role in leaf chlorophyll metabolism.

### 2.7. Overexpression of the CabHLH18-like Gene Promotes Chlorophyll Degradation

**Figure 7:**
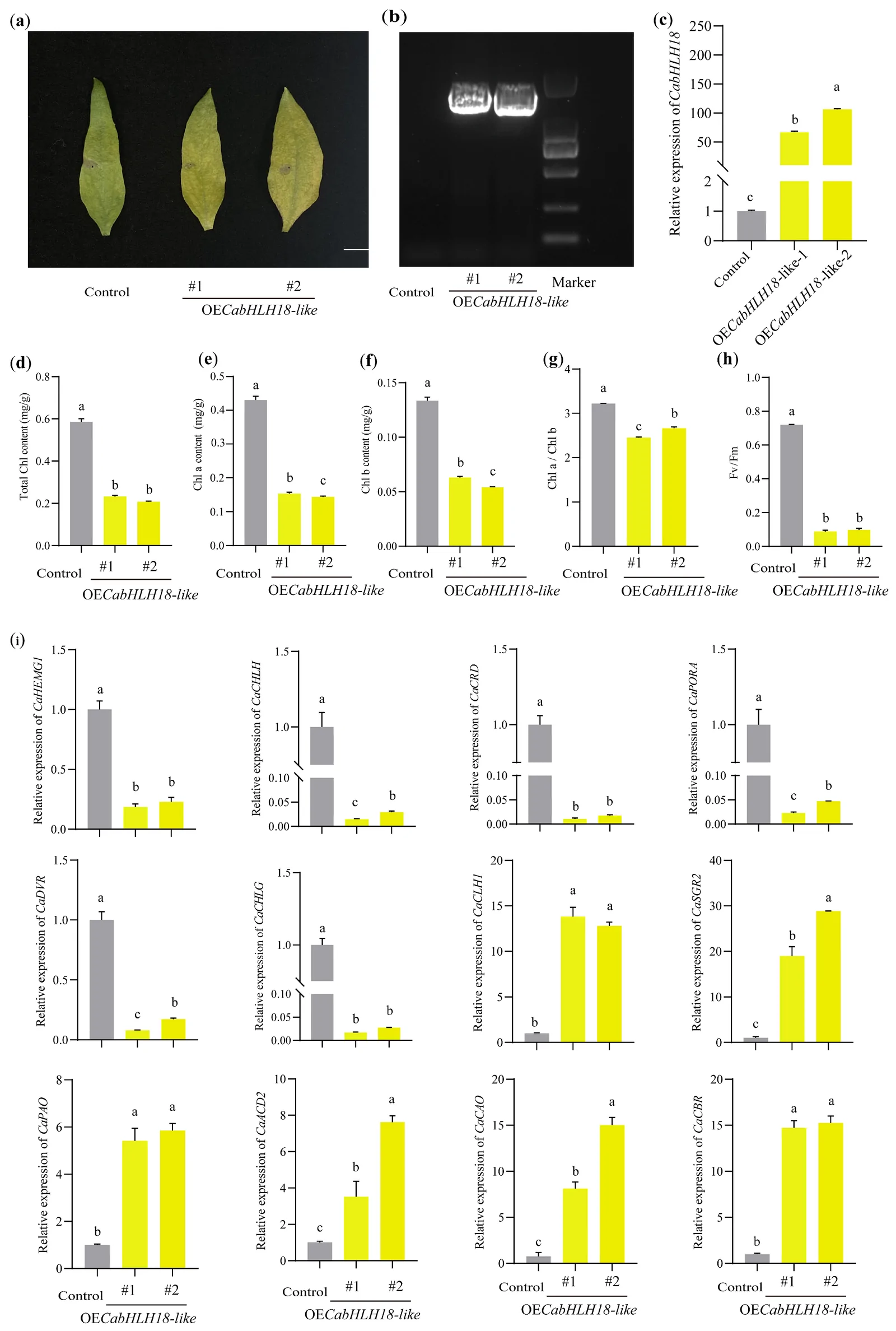
Transient overexpression of the *CabHLH18-like* gene induced leaf yellowing in pepper. (**a**) Phenotypic comparison of pepper leaves infiltrated with *Agrobacterium tumefaciens* harboring pCambia1300-*CabHLH18-like*-GFP (OE*CabHLH18-like*) or empty vector pCambia1300-GFP (control). Bar = 1 cm. (**b**) PCR-based detection of the *CabHLH18-like* transiently overexpressing leaves in control and *CabHLH18-like* transiently overexpressing leaves. M, 2000 bp DNA ladder. (**c**) Relative expression level of the *CabHLH18-like* gene in control and OE*CabHLH18-like* plants determined by qRT-PCR. (**d**–**h**) Total chl content (**d**), chl a content (**e**), chl b content (**f**), chl a/chl b (**g**) and maximum quantum yield of photosystem II (*Fv*/*Fm*) (**h**) in control and OE*CabHLH18-like* plants. (**i**) Expression profiles of chlorophyll biosynthesis (*HEMG1*, *CHLH*, *CRD*, *PORA*, *DVR*, *CHLG*), cycle (*CAO*, *CBR*) and degradation (*CLH1*, *SGR2*, *PAO*, *ACD2*) genes in control and *CabHLH18-like* overexpressed transcription leaves. *CaActin* served as the internal reference gene. Mean ± SD from three independent biological replicates. Letters above the bars indicate statistically significant differences determined by one-way ANOVA (*p* < 0.05).

To explore the effect of the *CabHLH18-like* gene on leaf coloration, a transient overexpression assay was performed. Ectopic overexpression of the *CabHLH18-like* gene in *Nicotiana benthamiana* caused pronounced leaf yellowing and led to significant decreases in chlorophyll content and the *Fv*/*Fm* (Figure S3). Consistent with this observation, *Agrobacterium*-mediated transient overexpression of the *CabHLH18-like* gene in pepper leaves produced a similar chlorotic phenotype (Figure 7a–c). The total chlorophyll, chlorophyll a, chlorophyll b, chl a/chl b, and *Fv*/*Fm* were all significantly reduced in *CabHLH18-like*-overexpressing tissues compared to the controls (Figure 7d–h).

We further analyzed the expression profiles of genes involved in chlorophyll biosynthesis and metabolism. qRT-PCR analysis demonstrated that the transcript abundance of chlorophyll biosynthetic genes, including *HEMG1*, *CHLH*, *CRD*, *PORA*, *DVR*, and *CHLG*, were significantly downregulated in *CabHLH18-like*-overexpressing leaves, whereas chlorophyll cycle-related genes (*CAO*, *CBR*) and degradation-related genes (*CLH1*, *SGR2*, *PAO*, *ACD2*) were markedly upregulated compared with controls (Figure 7i). Taken together, these findings indicate that the *CabHLH18-like* gene negatively regulates chlorophyll content by concurrently suppressing chlorophyll biosynthesis and enhancing chlorophyll degradation, thereby contributing to the leaf yellowing phenotype.

### 2.8. Silencing of the CabHLH18-like Gene Enhances Chlorophyll Accumulation

**Figure 8:**
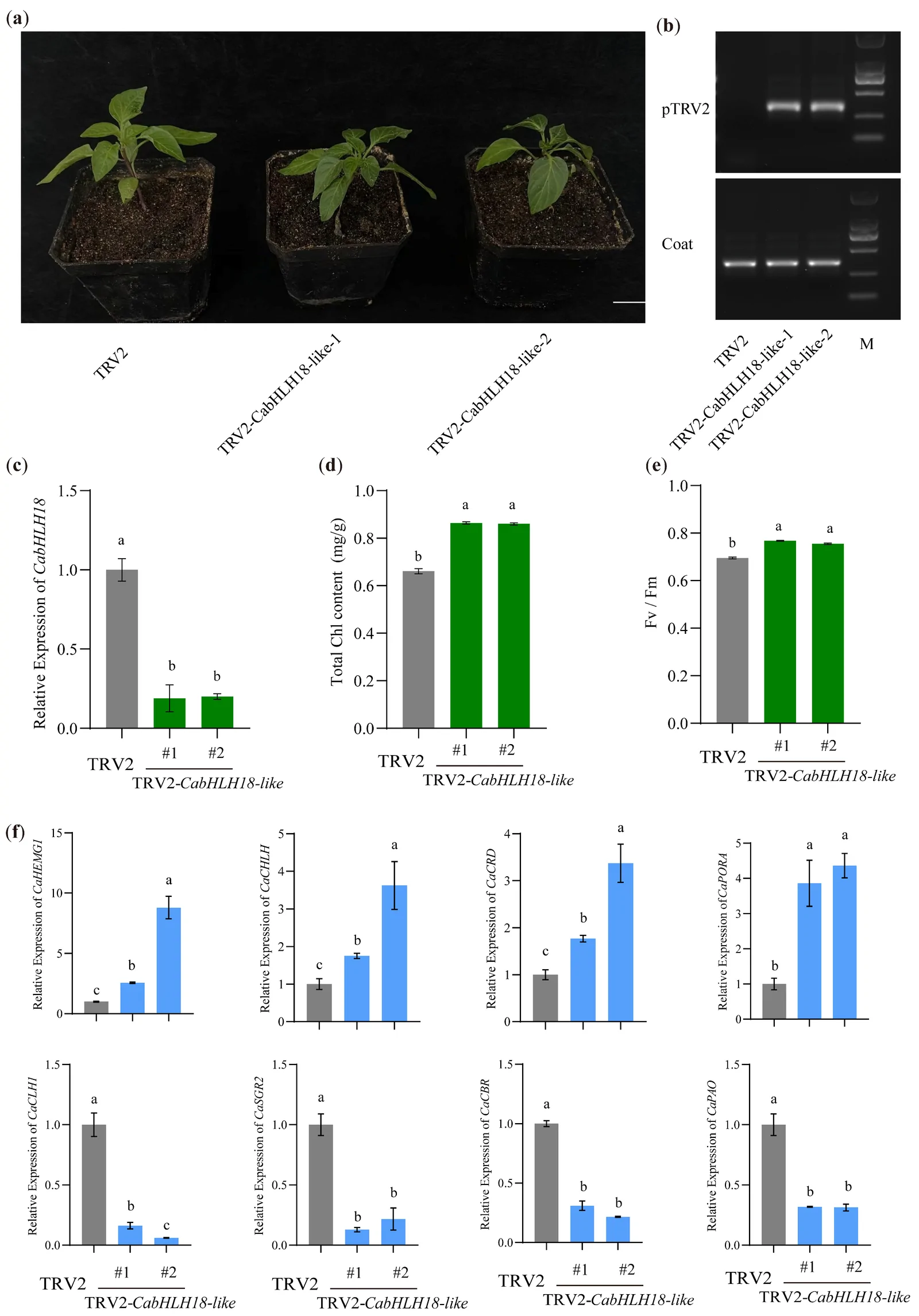
Silencing of the *CabHLH18-like* gene promoted chlorophyll accumulation in pepper. (**a**) Phenotypic comparison of NY pepper leaves infiltrated with *Agrobacterium tumefaciens* harboring pTRV2 empty vector (TRV2) or pTRV2-*CabHLH18-like* lines at six weeks post-infiltration. Scale bar = 2 cm. (**b**) The presence of the tobacco rattle virus (TRV) coat protein (CP) sequence in TRV2 and TRV2:*CabHLH18-like* plants was assessed by RT-PCR. M, 2000 bp DNA ladder. (**c**) Relative expression level of the *CabHLH18-like* gene in TRV2 and TRV2:*CabHLH18-like* plants determined by qRT-PCR. (**d**,**e**) Total chlorophyll content (**d**) and maximum quantum yield of photosystem II (*Fv*/*Fm*) (**e**) in TRV2 and TRV2:*CabHLH18-like* plants. (**f**) Expression profiles of chlorophyll biosynthesis (*HEMG1*, *CHLG*, *CRD*, *PORA*), cycle (*CBR*) and degradation (*CLH1*, *SGR2*, *PAO*) genes in TRV2 and TRV2:*CabHLH18-like* plants. *CaActin* served as the internal reference gene. Mean ± SD from three independent biological replicates. Letters above the bars indicated statistically significant differences determined by one-way ANOVA (*p* < 0.05).

Further, the *CabHLH18-like* gene was silenced in NY pepper through a tobacco rattle virus-based VIGS strategy. When the white phenotype appeared in the positive control (*PDS*-silenced) plants, both pTRV2 empty vector and pTRV2-*CabHLH18-like* lines were subjected to phenotypic observation and molecular characterization (Figure S4). Compared with pTRV2 control plants, pTRV2-*CabHLH18-like* plants exhibited markedly greener leaves (Figure 8a). RT-PCR confirmed the presence of TRV2 in NY plants (Figure 8b). qRT-PCR revealed an −80% reduction in *CabHLH18-like* transcript levels in the silenced lines (Figure 8c).

The total chlorophyll content and *Fv*/*Fm* ratio were significantly higher in pTRV2-*CabHLH18-like* plants than in pTRV2 controls (Figure 8d,e). qPCR analysis showed that chlorophyll biosynthesis genes, including *HEMG1*, *CHLG*, *CRD*, and *PORA*, were significantly upregulated in pTRV2-*CabHLH18-like* plants, whereas chlorophyll cycle-related gene (*CBR*) and the degradation genes (*CLH1*, *SGR2*, *PAO*) were markedly downregulated compared to pTRV2 controls (Figure 8f). These findings indicate that the *CabHLH18-like* gene negatively regulates chlorophyll accumulation in pepper, likely by coordinately repressing chlorophyll biosynthesis and promoting chlorophyll degradation pathways.

## 3. Discussion

Leaf color is a critical trait in plant growth and development, influencing photosynthetic efficiency and providing a visible indicator of environmental stress. The mechanisms that govern leaf color formation are relatively well characterized and primarily involve chlorophyll biosynthesis and metabolic pathways, making this a major focus in plant biology. The yellow leaf phenotype represents a common class of mutations that occur at high frequency, are easily recognized, and typically have non-lethal effects. These mutations often arise during early developmental stages and can be stably inherited. However, despite the economic importance of pepper as a specialty vegetable crop, the genetic mechanisms underlying yellow leaf phenotype in this species remain poorly characterized.

The basic helix–loop–helix (bHLH) transcription factor family has been reported to take part in leaf color regulation. In tomato, *SlPRE5* negatively regulated chlorophyll accumulation through repressing chloroplast development and photosynthesis-related gene expression [23]. *AtPIF4* and *AtPIF5* promote leaf senescence under dark conditions by directly activating *ORE1*, *EIN3*, and *ABI5*, thereby forming coherent feed-forward loops that integrate ethylene and ABA signaling [24], while *AtPIF4* also directly binds to the *NYE1* promoter to induce chlorophyll degradation [25]. *AtPIF4* also directly binds to the E-box motifs in the promoters of *GLK1* and *GLK2* to suppress their transcription, while at the same time activating *NYE1* and *SGR1* to accelerate chlorophyll degradation [26]. The *CabHLH18-like* gene is a member of the basic helix–loop–helix (bHLH) family, a major class of plant regulatory proteins involved in diverse developmental and metabolic pathways. In the previous study, CabHLH18 was shown to be involved in enhancing pepper tolerance to waterlogging stress [27]. Our phylogenetic analysis revealed that *CabHLH18-like* protein clusters have orthologs from *Solanum lycopersicum* and *Capsicum chacoense*, indicating strong evolutionary conservation within the Solanaceae family. Subcellular localization and tissue-specific expression analyses confirmed its nuclear localization and predominant expression in leaves, which is consistent with its predicted function as a transcriptional regulator of leaf pigmentation. Transient overexpression of the *CabHLH18-like* gene in both *Nicotiana benthamiana* and pepper leaves caused marked leaf yellowing and was accompanied by substantial decreases in total chlorophyll content and the *Fv*/*Fm*. Conversely, virus-induced gene silencing (VIGS) of the *CabHLH18-like* gene enhanced chlorophyll accumulation and produced greener leaves relative to controls. These reciprocal transient overexpression and virus-induced gene silencing (VIGS) phenotypes identify the *CabHLH18-like* gene as a positive regulator of chlorophyll degradation. However, *CabHLH18-like* overexpression decreases both chlorophyll a and chlorophyll b and therefore does not fully reproduce the chlorophyll profile of the natural NY material. This observation suggests that the altered chlorophyll a/b ratio in NY may also involve additional regulatory differences.

Leaf coloration is primarily determined by chlorophyll content, which is controlled by both biosynthetic and degradation pathways. In rice, the NAC transcription factor *OsNAP* positively regulates leaf senescence by directly activating chlorophyll degradation genes such as *OsSGR* and *OsNYC1*, and it also fine-tunes ABA biosynthesis through feedback regulation [28]. *AtbHLH18* negatively regulates iron uptake through *FIT* destabilization and subsequent repression of *IRT1*/*FRO2*, thereby indirectly repressing chlorophyll biosynthesis and promoting leaf chlorosis [29]. In Arabidopsis, the bHLH transcription factor *AtMYC2* integrates jasmonate signaling to coordinate chlorophyll biosynthesis and degradation during stress-induced leaf yellowing [30]. The AP2/ERF transcription factor *SlDEAR1* in tomato acts as a multifunctional regulator that activates chlorophyll biosynthesis gene *SlPOR1* and represses the degradation gene *SlSGR1* via TPL2-HDA1/3-mediated co-repressor complex, thereby promoting chlorophyll accumulation during fruit development [31]. Similarly, the *CabHLH18-like* gene might exert dual regulatory roles in chlorophyll accumulation through modulating the expression of genes related to both chlorophyll biosynthesis and degradation. In transcript-overexpressing leaves of the *CabHLH18-like* gene, chlorophyll biosynthetic genes, including *HEMG1*, *CHLH*, *CRD*, *PORA*, *DVR*, and *CHLG*, were downregulated, and these genes encode enzymes that catalyze early to late steps of the porphyrin and chlorophyll biosynthetic pathways, whereas the chlorophyll degradation and cycling genes, *CLH1*, *SGR2*, *PAO*, *ACD2*, *CAO*, and *CBR*, were markedly upregulated. This indicates accelerated chlorophyll degradation in the *CabHLH18-like*-overexpressing plants. We also observed this phenotype in the *CabHLH18-like*-silenced pants. After silencing of the *CabHLH18-like* gene, the chlorophyll degradation genes expressed lower while the chlorophyll biosynthetic genes expressed higher, and then chlorophyll content increased. These results suggest that the transcription factor *CabHLH18-like* gene may directly affect chlorophyll accumulation by modulating both chlorophyll biosynthesis and degradation pathways.

Transcription factors primarily regulate the transcription of genes through binding to core cis-elements within their promoters, thereby modulating chlorophyll synthesis and degradation. Under heat stress, the bZIP factors *ABF2/3/4* act as master regulators and directly activate the *SGR1* and *NYC1* promoters to accelerate chlorophyll degradation [32]. In a natural yellowing mutant of *Camellia chekiangoleosa*, multiple transcription factors, including ERF, WRKY, MYB, and TGA, were identified as potential regulators of the *CLH* gene through co-expression network analysis, suggesting their involvement in chlorophyll metabolism during spontaneous leaf color variation [33]. In soybean, the GATA transcription factor *GmGATA58* directly binds to the promoters of the chlorophyll biosynthetic genes *GmCHLI1*, *GmCHLH1*, and *GmCHLH3*, activating their transcription and promoting chlorophyll accumulation [34]. In rice, the bHLH transcription factor *OsPIL11* interacted with the promoter of *YGL3* to activate its transcription, thereby promoting chloroplast development and chlorophyll synthesis in a natural yellow leaf mutant [35]. Collectively, these studies show that transcription factors individually or cooperatively regulate chlorophyll biosynthesis and degradation to shape plant development and mediate responses to environmental signals. In this study, transient overexpression of *CaCLH1* in pepper caused pronounced leaf yellowing and reduced chlorophyll content. Furthermore, *CaCLH1* was identified as a direct downstream target of the *CabHLH18-like* gene. Y1H, LUC, and EMSA assays indicated that the *CabHLH18-like* gene could directly bind the promoter of *CaCLH1* (5′-CANNTG-3′) and activate its transcription. This was consistent with the leaf yellowing phenotype observed in *CabHLH18-like*-overexpressed plants. These results imply that the *CabHLH18-like* gene may participate in chlorophyll degradation by directly regulating *CaCLH1* expression. Taken together, the data suggest that *CaCLH1* is a direct downstream target of the CabHLH18-like gene and contributes to CabHLH18-like-mediated regulation of leaf yellowing in pepper.

Chlorophyllase (CLH) is a key enzyme in the chlorophyll degradation pathway and is highly conserved across plant species. CLH is generally classified into two groups: Group I, which responds to environmental and hormonal signals, and Group II, which contributes to chlorophyll homeostasis. CLH is highly expressed during the seedling stage and associated with photosystem II (PSII) repair. In Arabidopsis, CLH1 is localized to chloroplasts and protects young leaves from photodamage by promoting D1 protein turnover [36]. In tea, overexpression of *CsCLH1* causes light green leaves, while silencing of *CsCLH1* showed dark green [37]. Furthermore, transcriptionally upregulating CLH accelerates chlorophyll breakdown and aggravates symptom development in infected cucurbit tissues [38]. These findings indicate that CLH plays a core role in chlorophyll degradation. In this study, we identified a natural stably yellow leaf natural variant (NY) in pepper. TEM analysis revealed no obvious abnormalities in chloroplast ultrastructure compared with green leaves, suggesting that the phenotype is not caused by impaired chloroplast development. Transcriptome analysis indicated that genes associated with chlorophyll biosynthesis and metabolism were differentially expressed, with *CaCLH1* showing notable elevated expression in NY. Functional analyses further indicated that overexpression of *CaCLH1* enhanced chlorophyll degradation, leading to the yellow phenotype. These findings support the conclusion that *CaCLH1* also acts as a positive regulator of chlorophyll degradation in pepper.

Since ZX and NY represent sweet pepper lines with different genetic backgrounds, the differentially expressed genes identified in this study may include both leaf-color-related candidate genes and genes whose expression differences arise from general genetic background effects. Therefore, caution should be exercised when interpreting the transcriptomic data to distinguish leaf-color-specific candidate genes from background-driven expression variations. Future studies will employ near-isogenic lines (NILs) or F_2_ segregating populations to further validate the specific expression patterns of leaf color candidate genes and to exclude the confounding influence of genetic background.

In conclusion, we identified a natural yellow leaf phenotype in pepper that is associated with reduced chlorophyll content. *CaCLH1* was validated as a positive regulator of leaf yellowing. Through screening of upstream regulatory genes, bHLH transcription factor CabHLH18-like gene directly bound to the promoter of *CaCLH1* to activate its transcription. Overexpression and silencing of the *CabHLH18-like* gene clarified that it upregulates chlorophyll degradation in pepper. The chlorophyll biosynthesis genes were downregulated, whereas chlorophyll-cycle and chlorophyll-degradation genes were upregulated in CabHLH18-like transcript-overexpressing leaves. In summary, *CaCLH1* is a direct downstream target of the *CabHLH18-like* gene, and their regulatory interplay contributes to chlorophyll turnover in pepper. This study provides a viable target for molecular breeding of varieties with customized pigmentation.

## 4. Materials and Methods

### 4.1. Plant Materials and Treatments

Sweet pepper cultivars, ZIXINGJIAO (ZX) and NAIYOUJIAO (NY), were provided by the Hebei Academy of Agricultural and Forestry Sciences and grown in a controlled growth chamber. Plants were grown under a 16 h light (28 °C) and 8 h dark (22 °C) photoperiod. *Nicotiana benthamiana* plants were cultivated under the same conditions for subsequent experiments. Leaf samples were collected at −80 °C.

Tissue samples, including roots, stems, leaves, and flowers, were collected from 8-week-old NY pepper. All samples were immediately stored at −80 °C for further experimentation.

The materials, plasmids, and bacterial strains employed in this experiment were preserved and supplied by our institute.

### 4.2. RNA Extraction and Transcriptome Analysis

Total RNA was extracted according to previously described protocols [39]. Transcriptome sequencing was performed by Metware Biotechnology Co., Ltd. (Wuhan, China). After quality filtering, trimming, and sequence alignment, gene expression levels were calculated and represented by FPKM. The reference genome was http://ted.bti.cornell.edu/ftp/pepper/genome/Zhangshugang/ (accessed on 26 May 2026). Reads were aligned with HISAT2 v2.2.1 and quantified using featureCounts v2.0.3. Differential expressions were analyzed with DESeq2 v1.22.1 in R Programming Language (https://www.r-project.org/), based on three biological replicates per sample. DEGs were defined as FDR < 0.05 and |log_2_FC| ≥ 1. GO and KEGG pathway enrichment analyses were conducted using hypergeometric distribution tests.

### 4.3. RNA Extraction and Quantitative Real-Time PCR (qRT-PCR)

Total RNA was extracted based on the Trizol-based method following the protocol (Jiangsu Cowin Biotech Co., Ltd., Taizhou, Jiangsu, China, CW0580) [40]. For cDNA synthesis, 1 μg of total RNA was subjected to reverse transcription with the HiScript III RT SuperMix (Jiangsu Cowin Biotech Co., Ltd., Taizhou, Jiangsu, China, CW2569). qRT-PCR was conducted using the UltraSYBR Mixture (Jiangsu Cowin Biotech Co., Ltd., Taizhou, Jiangsu, China, CW0957). The 20 μL reaction system contained four parts: 10 μL of 2× UltraSYBR Mixture, 0.4 μL of each gene-specific primer (10 μM), 2 μL of diluted cDNA template, and 7.2 μL of nuclease-free water. The qRT-PCR program followed 95 °C for 30 s; 40 cycles of 95 °C for 10 s; and 60 °C for 30 s. Primers used are listed in Table S1. *CaActin* and *NbActin* served as the internal standard. The expression levels of genes were calculated according the 2^−ΔΔCt^ method [41]. Three independent biological replicates were performed, with each replicate analyzed in technical triplicate.

### 4.4. Transient Overexpression via Agrobacterium-Mediated Infiltration in Tobacco and Pepper

The full-length coding sequence of the *CabHLH18-like* gene and *CaCLH1* was extracted from the cDNA of the NY pepper and constructed into the pCambia1300-GFP binary vector to generate the fusion construct pCambia1300-*CabHLH18-like*-GFP and pCambia1300-*CaCLH1*-GFP, respectively. The recombinant plasmid was transformed into GV3101 competent cells. For transient expression, *Agrobacterium* with the *CabHLH18-like* gene or *CaCLH1* was resuspended in infiltration buffer and adjusted to OD_600_ = 0.6. The bacterial suspension was infiltrated into the leaves of green pepper and tobacco using a needleless syringe. Plants were maintained under controlled conditions (16 h light/8 h dark, 22 °C, 60% relative humidity), and leaf color changes were observed. Empty plasmid (pCambia1300-GFP) infiltration served as negative controls.

### 4.5. Silencing of the CabHLH18-like Gene in Pepper

Virus-induced gene silencing was performed according to a previously described protocol [42]. A 300 bp fragment of the *CabHLH18-like* gene was amplified and cloned into the pTRV2 vector via homologous recombination to generate pTRV2-*CabHLH18-like* (sequence listed in Table S1). The recombinant plasmid was introduced into GV3101. For VIGS assays, *Agrobacteria* with pTRV1 and pTRV2 (or pTRV2-*CabHLH18-like*) were individually grown overnight at 28 °C. The bacterial suspensions were collected and resuspended with infiltration buffer to OD_600_ = 0.6. Equal volumes of pTRV1 and pTRV2 (or pTRV2-*CabHLH18-like*) suspensions were mixed (1:1, *v*/*v*). The mixture was infiltrated into the cotyledons of two-week-old NY pepper seedlings using a needleless syringe. Inoculated plants were maintained in the dark at 22 °C for 24 h and then grown in a plant chamber (16 h light/8 h dark, 22 °C). After six weeks of infiltration, reverse-transcribed cDNA was collected and the presence of the TRV coat protein (CP) was detected using RT-PCR (primers listed in Table S1). The silencing efficiency of the *CabHLH18-like* gene was evaluated by qRT-PCR using *CaActin* as the internal loading.

### 4.6. Yeast One-Hybrid (Y1H) Assay

The open reading frame of the *CabHLH18-like* gene was cloned into the pGADT7 vector. A 620 bp fragment of the *CaCLH1* promoter spanning −1516 to −896 bp was cloned into the pAbAi vector. Then, pAbAi-*proCaCLH1* was linearized with BstBI and integrated into the Y1H Gold yeast strain.

To determine the optimal concentration of aureobasidin A (AbA), pAbAi-*proCaCLH1* integrant was cultured on SD/-Ura medium supplemented with a gradient of AbA (0, 100, 200, 300, 400, 500, and 1000 ng/mL). The minimal concentration that completely suppressed background growth of the bait strain was determined to be 400 ng/mL, which was used for subsequent interaction screening.

For Y1H assays, the pGADT7-*CabHLH18-like* prey construct and the empty pGADT7 negative control were transformed into competent Y1H Gold cells with the pAbAi-*proCaCLH1* bait. To assess protein–DNA interactions, co-transformants were spotted onto the medium of SD/-Leu and SD/-Leu supplemented with 400 ng/mL AbA in 10-fold serial dilutions (100, 10^−1^, 10^−2^). Growth on AbA-containing medium indicated specific binding of the *CabHLH18-like* gene to the *CaCLH1* promoter.

### 4.7. Dual-Luciferase Reporter Assay

The open reading frame of the *CabHLH18-like* gene was constructed into the pGreenII 62-SK vector to generate the effector construct (35S:*CabHLH18-like*). A 1500 bp fragment of the *CaCLH1* promoter was amplified and inserted into the pGreenII 0800-LUC plasmid to generate the reporter construct (*CaCLH1pro*: LUC). All primers used are listed in Supplementary Table S1. The effector and reporter plasmids were individually transformed into *Agrobacterium* GV3101 (pSoup).

For transient expression, GV3101 cultures harboring the effector, reporter, or empty vector controls were resuspended in infiltration buffer and adjusted to OD_600_ = 0.8. Equal volumes of effector and reporter suspensions were mixed at a 9:1 ratio (effector:reporter). The mixtures were infiltrated into 4-week-old tobacco leaves. Infiltrated plants were maintained under 16 h light/8 h dark and 22 °C for 60 h. Then, infected plants were sprayed with 100 mM D-luciferin potassium salt (Promega Corporation, Madison, WI, USA, E1602) and incubated in darkness for 5 min. LUC and REN signals were observed using an imaging system (NightShade LB 985, Berthold Technologies GmbH & Co.KG, Bad Wildbad, Germany). For quantitative analysis, infected leaves were harvested and stored at −80 °C. The activity of firefly luciferase (LUC) and Renilla luciferase (REN) were measured sequentially following the Dual-Luciferase Reporter Assay System (Beijing Coolaber Technology Co., Ltd., Beijing, China, DL101) [43].

### 4.8. Electrophoretic Mobility Shift Assay (EMSA)

The *CabHLH18-like* ORF was directionally cloned into the multiple cloning site of pET-28a and then transformed into chemically competent *E. coli* BL21 (DE3). Cultures were treated with 0.5 mM IPTG and incubated at 16 °C for 16 h to accumulate the CabHLH18-like-His protein, which was later isolated using Ni-NTA agarose beads (Jiangsu Cowin Biotech Co., Ltd., Taizhou, Jiangsu, China, CW0894S) according to the manufacturer’s protocol [44].

For EMSA, a 24 bp double-stranded oligonucleotide probe containing the wild-type E-box cis-element (5′-CANNTG-3′) within the *CaCLH1* promoter was synthesized, with biotin at the 5′ end (Sangon Biotech, Shanghai, China). An unlabeled competitor probe and a mutated probe with the core motif substituted by TTTTT (5′-TTTTTT-3′) were synthesized as cold competitors and negative controls, respectively. For competition assays, 10× and 100× of an unlabeled probe were added. The biotin-labeled DNA–protein complexes were transferred to a positively charged nylon membrane and detected using the Chemiluminescent EMSA Kit (Shanghai Beyotime Biotechnology Co., Ltd., Shanghai, China, GS009) following the manufacturer’s instructions [45].

### 4.9. Phylogenetic Tree Construction of the CabHLH18-like Gene and Its Homologs

The total amino acid sequences of the CabHLH18-like gene and its homologs from *Capsicum annuum*, *Capsicum chacoense*, *Solanum demissum*, and other plant species were aligned using ClustalW implemented in MEGA X (v10.2.6) (Table S2). Phylogenetic trees were constructed using the neighbor-joining (NJ) method with 1000 bootstrap replicates. Bootstrap values were calculated from 1000 replicates to assess the reliability of each node. The resulting trees were visualized and annotated using iTOL v6.

### 4.10. Subcellular Localization

The open reading frame (ORF) of the *CabHLH18-like* gene was cloned into the pRI101-GFP vector, generating the pRI101-*CabHLH18-like*-GFP fusion construct. The recombinant plasmid was verified by PCR and Sanger sequencing and then introduced into the *Agrobacterium tumefaciens* strain. For transient expression, Agrobacterium cultures harboring the fusion construct were resuspended in infiltration buffer and adjusted to OD_600_ of 0.6. The bacterial suspension was infiltrated into the leaves of 4-week-old *Nicotiana benthamiana*. After infiltrating, the plants were grown in darkness (22 °C, 24 h) and then transferred to standard growth conditions (16 h light/8 h dark, 22 °C) until 60 h. GFP signals were captured on a Leica TCS SP8 confocal microscope (Leica Microsystems IR GmbH, Wetzlar, Germany) under 488 nm excitation, with emission collected between 500 and 530 nm.

### 4.11. Determination of Chlorophyll Content

Chlorophyll was extracted following the protocol of Arnon (1949) with minor modifications [46]. Briefly, 0.2 g of fresh sample was homogenized in 2 mL of 80% (*v*/*v*) acetone and incubated in darkness (4 °C, 24 h). Then, the mixture was centrifuged at 12,000× *g*, 10 min under 4 °C. The supernatant was subjected at 645 and 663 nm using a microplate reader (Tecan Austria GmbH, Grödig, Austria).

Total Chl content (mg/g) = (20.29A_645_ + 8.05A_663_) × V/(1000 × W)

Chl a content (mg/g) = (12.72A_663_ − 2.59A_645_) × V/(1000 × W)

Chl b content (mg/g) = (22.88A_645_ − 4.67A_663_) × V/(1000 × W)

V: the total volume of the extraction solution (mL); W: the fresh weight of the leaf tissue (g).

### 4.12. Determination of Fv/Fm

The *F_V_*/*F_m_* was measured following the protocol [47]. In brief, leaves were dark-adapted for 30 min, and *F*_0_ was recorded under measuring light after signal stabilization. A saturation pulse (>6000 µmol·m^−2^·s^−1^, 0.3–1.0 s) was then applied to determine *F_m_*. PSII maximum photochemical efficiency (*F_V_*/*F_m_*) was calculated:

*F_V_*/*F_m_* = (*F_m_* − *F*_0_)/*F_m_*

### 4.13. Statistical Analyses

Statistical significance was determined by *t*-test or one-way ANOVA with Tukey’s test analysis using GraphPad Prism version 7.0 (GraphPad Software, San Diego, CA, USA). Significant differences were indicated by * *p* < 0.05, ** *p* < 0.01, or *** *p* < 0.001. Letters above the bars indicated statistically significant differences determined by one-way ANOVA (*p* < 0.05).

## 5. Conclusions

**Figure 9:**
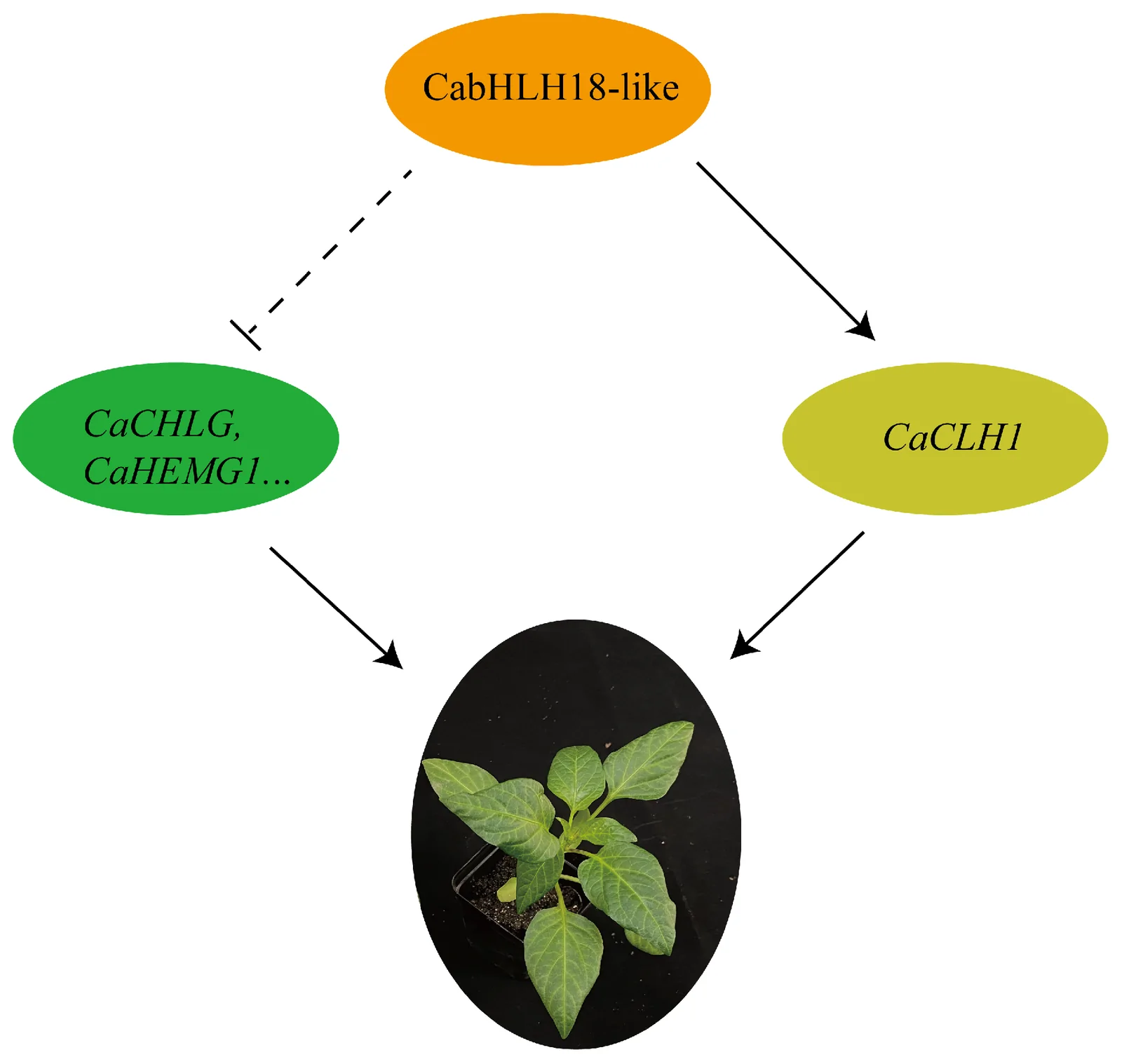
Summary mechanism diagram. In pepper, the CabHLH18-like gene may promote chlorophyll degradation by directly activating *CaCLH1* transcription, while *CabHLH18-like* overexpression was associated with reduced expression of chlorophyll biosynthesis genes, ultimately leading to leaf yellowing.

In conclusion, this study identified a natural yellow leaf variant (NY) in pepper and demonstrated that *CaCLH1* overexpression drives leaf yellowing. We further showed that the transcription factor CabHLH18-like gene directly activates *CaCLH1* transcription by binding its promoter, thereby promoting chlorophyll degradation. Functional analyses confirmed that the *CabHLH18-like* gene positively regulates chlorophyll metabolism: silencing increases chlorophyll content, whereas overexpression decreases it. Together, these findings indicate that *CaCLH1* is a direct downstream target of the *CabHLH18-like* gene and contributes to *CabHLH18-like*-mediated regulation of leaf yellowing in pepper, thereby advancing our understanding of the molecular networks that control leaf color variation (Figure 9). This work provides a genetic target for germplasm improvement in pepper.

## Data Availability

The RNA-seq data can be found in the NCBI database with the accession code PRJNA1458070.

## Supplementary Materials

The following supporting information can be downloaded at: https://www.mdpi.com/article/10.3390/plants15172627/s1. Supplementary Figure S1. Transmission electron microscopy (TEM) observation of chloroplast ultrastructure between ZX and NY; Supplementary Figure S2. GO analysis of DEGs between NY and ZX; Supplementary Figure S3. Transient overexpression of the CabHLH18-like gene induces leaf yellowing in *Nicotiana benthamiana*; Supplementary Figure S4. TRV2-*CaPDS* phenotype in pepper; Supplementary Figure S5. Original gel electrophoresis images for DNA-level detection of the CabHLH18-like gene; Supplementary Figure S6. Original gel electrophoresis images for DNA-level detection of pTRV2-*CabHLH18-like lines*; Supplementary Figure S7. Original gel electrophoresis images for detection of coat protein; Supplementary Table S1. Primers used for this study; Supplementary Table S2. Protein sequence of the CabHLH18-like gene and its homologs. Supplementary Table S3. Data used for RNA-seq analysis.

## References

[1] Kim S., Park M., Yeom S.-I., Kim Y.-M., Lee J.M., Lee H.-A., Seo E., Choi J., Cheong K., Kim K.-T. (2014). Genome Sequence of the Hot Pepper Provides Insights into the Evolution of Pungency in Capsicum Species. Nat. Genet..

[2] Chen W., Wang X., Sun J., Wang X., Zhu Z., Ayhan D.H., Yi S., Yan M., Zhang L., Meng T. (2024). Two Telomere-to-Telomere Gapless Genomes Reveal Insights into Capsicum Evolution and Capsaicinoid Biosynthesis. Nat. Commun..

[3] Tanaka R., Tanaka A. (2007). Tetrapyrrole Biosynthesis in Higher Plants. Annu. Rev. Plant Biol..

[4] Reinbothe S., Reinbothe C. (1996). Regulation of Chlorophyll Biosynthesis in Angiosperms. Plant Physiol..

[5] Tanaka R., Kobayashi K., Masuda T. (2011). Tetrapyrrole Metabolism in Arabidopsis Thaliana. Arab. Book.

[6] Walker C.J., Willows R.D. (1997). Mechanism and Regulation of Mg-Chelatase. Biochem. J..

[7] Heyes D.J., Hunter C.N. (2005). Making Light Work of Enzyme Catalysis: Protochlorophyllide Oxidoreductase. Trends Biochem. Sci..

[8] Hedtke B., Alawady A., Chen S., Börnke F., Grimm B. (2007). HEMA RNAi Silencing Reveals a Control Mechanism of ALA Biosynthesis on Mg Chelatase and Fe Chelatase. Plant Mol. Biol..

[9] Kumar A., Soll D. (2000). Antisense HEMA1 RNA Expression Inhibits Heme and Chlorophyll Biosynthesis in Arabidopsis. Plant Physiol..

[10] Zhang H., Li J., Yoo J.-H., Yoo S.-C., Cho S.-H., Koh H.-J., Seo H., Paek N.-C. (2006). Rice Chlorina-1 and Chlorina-9 Encode ChlD and ChlI Subunits of Mg-Chelatase, a Key Enzyme for Chlorophyll Synthesis and Chloroplast Development. Plant Mol. Biol..

[11] Huang Y.-S., Li H.-M. (2009). Arabidopsis CHLI2 Can Substitute for CHLI1. Plant Physiol..

[12] Zhao Z., Li J., Zhao X., Wu S., Zhang Y., Liu Z., Tan C. (2025). Mutation of a Magnesium Chelatase *BrCHLD* Affects the Chlorophyll Content and Magnesium Chelatase Activity in Chinese Cabbage. Plant Physiol. Biochem..

[13] Yasumura Y., Moylan E., Langdale J. (2005). A Conserved Transcription Factor Mediates Nuclear Control of Organelle Biogenesis in Anciently Diverged Land Plants. Plant Cell.

[14] Pan Y., Bradley G., Pyke K., Ball G., Lu C., Fray R., Marshall A., Jayasuta S., Baxter C., Wijk R. (2013). Network Inference Analysis Identifies an APRR2-Like Gene Linked to Pigment Accumulation in Tomato and Pepper Fruits. Plant Physiol..

[15] Shin J., Kim K., Kang H., Zulfugarov I., Bae G., Lee D., Choi G. (2009). Phytochromes Promote Seedling Light Responses by Inhibiting Four Negatively-Acting Phytochrome-Interacting Factors. Proc. Natl. Acad. Sci. USA.

[16] Zhang T., Zhang R., Zeng X.-Y., Lee S., Ye L.-H., Tian S.-L., Zhang Y.-J., Busch W., Zhou W.-B., Zhu X.-G. (2024). GLK Transcription Factors Accompany ELONGATED HYPOCOTYL5 to Orchestrate Light-Induced Seedling Development in Arabidopsis. Plant Physiol..

[17] Gias Uddin J., Zhuo T., Li X., Wu X., Wu Z., Habiba, Ren Y., Miao Y. (2025). Overexpressing BrWRKY22 Delays Flowering and Leaf Senescence via Inhibition of GA Biosynthesis in Brassica Rapa. Plants.

[18] Chen X., Gao Z., Yu Z., Ding Q., Qian X., Zhang C., Zhu C., Wang Y., Zhang C., Li Y. (2024). BcWRKY53 Promotes Chlorophyll Biosynthesis and Cold Tolerance of Non-Heading Chinese Cabbage under Cold Stress. Plant Physiol. Biochem..

[19] Chen H., Ji H., Huang W., Zhang Z., Zhu K., Zhu S., Chai L., Ye J., Deng X. (2024). Transcription Factor CrWRKY42 Coregulates Chlorophyll Degradation and Carotenoid Biosynthesis in Citrus. Plant Physiol..

[20] Tsuchiya T., Ohta H., Okawa K., Iwamatsu A., Shimada H., Masuda T., Takamiya K. (1999). Cloning of Chlorophyllase, the Key Enzyme in Chlorophyll Degradation: Finding of a Lipase Motif and the Induction by Methyl Jasmonate. Proc. Natl. Acad. Sci. USA.

[21] Li W.-F., Liu J., Hu T.-Y., Hou Y.-J., Ma Z.-H., Feng T., Guo Z.-G., Mao J., Chen B.-H. (2025). Genome-Wide Survey of Chlorophyllase (CLH) Gene Family in Seven Rosaceae and Functional Characterization of *MdCLH1* in Apple (*Malus domestica*) Leaf Photosynthesis. Plant Physiol. Biochem..

[22] Hao Q., Li T., Lu G., Wang S., Li Z., Gu C., Kong F., Shu Q., Li Y. (2025). Chlorophyllase (PsCLH1) and Light-Harvesting Chlorophyll *a*/*b* Binding Protein 1 (PsLhcb1) and PsLhcb5 Maintain Petal Greenness in *Paeonia suffruticosa* ‘Lv Mu Yin Yu’. J. Adv. Res..

[23] Li J., Gong J., Zhang L., Shen H., Chen G., Xie Q., Hu Z. (2022). Overexpression of SlPRE5, an Atypical bHLH Transcription Factor, Affects Plant Morphology and Chlorophyll Accumulation in Tomato. J. Plant Physiol..

[24] Sakuraba Y., Jeong J., Kang M.-Y., Kim J., Paek N.-C., Choi G. (2014). Phytochrome-Interacting Transcription Factors PIF4 and PIF5 Induce Leaf Senescence in Arabidopsis. Nat. Commun..

[25] Song Y., Yang C., Gao S., Zhang W., Li L., Kuai B. (2014). Age-Triggered and Dark-Induced Leaf Senescence Require the bHLH Transcription Factors PIF3, 4, and 5. Mol. Plant.

[26] Pham V.N., Kathare P.K., Huq E. (2018). Phytochromes and Phytochrome Interacting Factors. Plant Physiol..

[27] Tian H., Fan G., Xiong X., Wang H., Zhang S., Geng G. (2024). Characterization and Transformation of the CabHLH18 Gene from Hot Pepper to Enhance Waterlogging Tolerance. Front. Plant Sci..

[28] Liang C., Wang Y., Zhu Y., Tang J., Hu B., Liu L., Ou S., Wu H., Sun X., Chu J. (2014). OsNAP Connects Abscisic Acid and Leaf Senescence by Fine-Tuning Abscisic Acid Biosynthesis and Directly Targeting Senescence-Associated Genes in Rice. Proc. Natl. Acad. Sci. USA.

[29] Cui Y., Chen C.-L., Cui M., Zhou W.-J., Wu H.-L., Ling H.-Q. (2018). Four IVa bHLH Transcription Factors Are Novel Interactors of FIT and Mediate JA Inhibition of Iron Uptake in Arabidopsis. Mol. Plant.

[30] Zhu X., Chen J., Xie Z., Gao J., Ren G., Gao S., Zhou X., Kuai B. (2015). Jasmonic Acid Promotes Degreening via MYC2/3/4- and ANAC019/055/072-Mediated Regulation of Major Chlorophyll Catabolic Genes. Plant J. Cell Mol. Biol..

[31] Pei Y., He X., Xue Q., Deng H., Xu W., Yang C., Wu M., Wang W., Tang W., Niu W. (2025). The Bifunctional Transcription Factor DEAR1 Oppositely Regulates Chlorophyll Biosynthesis and Degradation in Tomato Fruits. Plant Cell.

[32] Gao S., Gao J., Zhu X., Song Y., Li Z., Ren G., Zhou X., Kuai B. (2016). ABF2, ABF3, and ABF4 Promote ABA-Mediated Chlorophyll Degradation and Leaf Senescence by Transcriptional Activation of Chlorophyll Catabolic Genes and Senescence-Associated Genes in Arabidopsis. Mol. Plant.

[33] Huang B., Huang W., Liu Z., Peng Y., Qu Y., Zhou W., Huang J., Shu H., Wen Q. (2024). Cytological, Physiological, and Transcriptome Analysis of Leaf-Yellowing Mutant in *Camellia chekiangoleosa*. Int. J. Mol. Sci..

[34] Zhang C., Huang Y., Xiao Z., Yang H., Hao Q., Yuan S., Chen H., Chen L., Chen S., Zhou X. (2020). A GATA Transcription Factor from Soybean (Glycine Max) Regulates Chlorophyll Biosynthesis and Suppresses Growth in the Transgenic *Arabidopsis thaliana*. Plants.

[35] Chen W., Tang L., Li Q., Cai Y., Ahmad S., Wang Y., Tang S., Guo N., Wei R., Tang S. (2024). YGL3 Encoding an IPP and DMAPP Synthase Interacts with OsPIL11 to Regulate Chloroplast Development in Rice. Rice.

[36] Hu X., Khan I., Jiao Q., Zada A., Jia T. (2021). Chlorophyllase, a Common Plant Hydrolase Enzyme with a Long History, Is Still a Puzzle. Genes.

[37] Liu W., Liu S., Zhang K., Xie M., Sun H., Huang X., Zhang L., Li M. (2023). Chlorophyllase Is Transcriptionally Regulated by CsMYB308/CsDOF3 in Young Leaves of Tea Plant. Hortic. Plant J..

[38] Li Z., Tang Y., Lan G., Yu L., Ding S., He Z., She X. (2025). Cucumber Green Mottle Mosaic Virus Decreases Chlorophyll a Content in Cucurbit Crops by Upregulating the Key Gene in Chlorophyll Catabolic Pathway, Chlorophyllase 1. Plants.

[39] Chomczynski P., Sacchi N. (2006). The Single-Step Method of RNA Isolation by Acid Guanidinium Thiocyanate–Phenol–Chloroform Extraction: Twenty-Something Years On. Nat. Protoc..

[40] Sha G., Sun P., Kong X., Han X., Sun Q., Fouillen L., Zhao J., Li Y., Yang L., Wang Y. (2023). Genome Editing of a Rice CDP-DAG Synthase Confers Multipathogen Resistance. Nature.

[41] Livak K.J., Schmittgen T. (2001). Analysis of Relative Gene Expression Data Using Real-Time Quantitative PCR and the 2-DDCt Method. Methods.

[42] Zhou Y., Deng Y., Liu D., Wang H., Zhang X., Liu T., Wang J., Li Y., Ou L., Liu F. (2021). Promoting Virus Induced Gene Silencing of Pepper Genes by a Heterologous Viral Silencing Suppressor. Plant Biotechnol. J..

[43] Du C., Yun Y., Li W., Wu X., Liu X., Li M., Li Y., Wang S., Li W., He Q. (2026). A Natural Variation within Duplicated *AsWRKY49-D2* Drives the Subgenomic Functional Divergence of Homeologs in Salt Response of Allohexaploid Oats. J. Integr. Plant Biol..

[44] Kuang J., Xu Y., Liu Y., Zhang R., Li X., Wang Y., Jin Q. (2024). An NnSnRK1-Centered Regulatory Network of Shade-Induced Early Termination of Flowering in Lotus. Environ. Exp. Bot..

[45] Wang Y., Gong Q., Wu Y., Huang F., Ismayil A., Zhang D., Li H., Gu H., Ludman M., Fátyol K. (2021). A Calmodulin-Binding Transcription Factor Links Calcium Signaling to Antiviral RNAi Defense in Plants. Cell Host Microbe.

[46] Arnon D.I. (1949). Copper Enzymes in Isolated Chloroplasts. Polyphenoloxidase in Beta Vulgaris. Plant Physiol..

[47] Maxwell K., Johnson G.N. (2000). Chlorophyll Fluorescence—A Practical Guide. J. Exp. Bot..

